# Supplementary material for: Harnessing metastability for grain size control in multiprincipal element alloys during additive manufacturing
Source: Nat Commun. 2025 Feb 12;16:1446. doi: 10.1038/s41467-025-56616-0 (PMC11822122; doi:10.1038/s41467-025-56616-0)
Supplement: Supplementary file 1 — Supplementary Information [file 41467_2025_56616_MOESM1_ESM.pdf]

Harnessing metastability for grain size control in multiprincipal element alloys during additive manufacturing

Authors: Akane Wakai[1], Jenniffer Bustillos[1], Noah Sargent[2], Jamesa Stokes[3], Wei Xiong[2], Timothy M. Smith[3], Atieh Moridi[1]*

Affiliations
[1] Department of Mechanical and Aerospace Engineering, Cornell University, Ithaca, NY, USA
[2] Department of Mechanical Engineering and Materials Science, University of Pittsburgh, Pittsburgh, PA, USA
[3] NASA Glenn Research Center, Cleveland, OH, USA

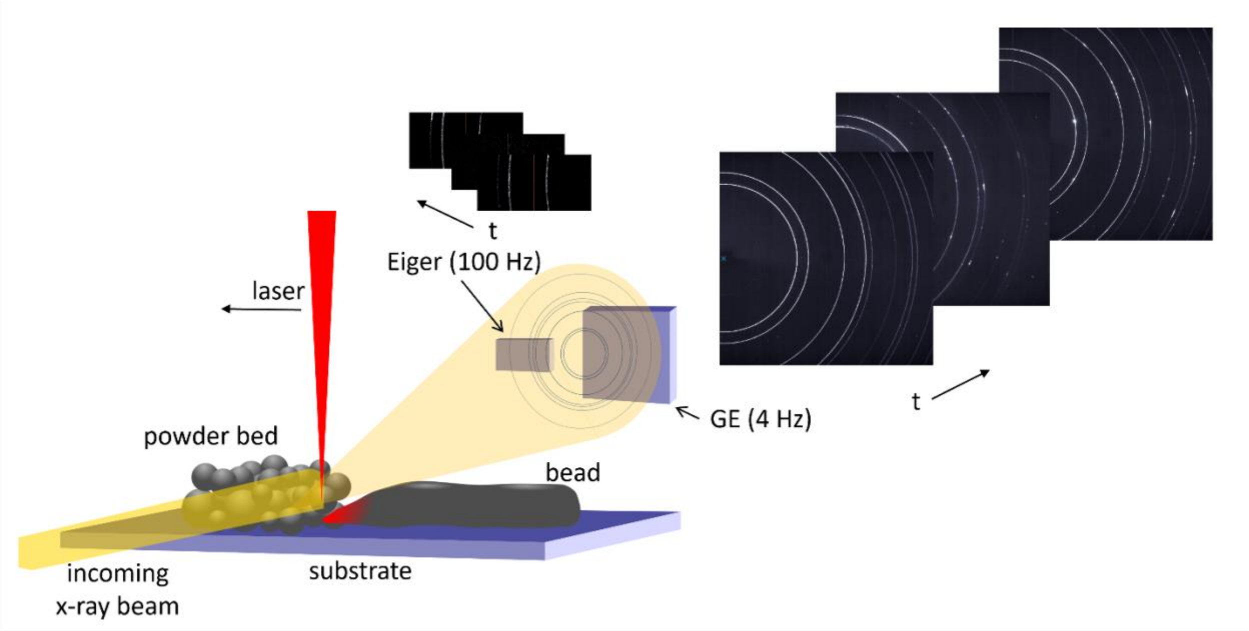

Supplementary Fig. 1. Schematic representation of the custom setup integrated at CHESS. An enclosure (not pictured) maintains an inert atmosphere while the laser beam rasters over a powder bed to simulate an AM environment. Although the powder preparation follows a powder-bed style setup, the scanning speed used in the study results in cooling rates characteristic of a DED processing condition. The transmitted XRD pattern is captured by two detectors (CdTe Eiger 500k area detector and a GE 41-RT area detector) at 100 Hz and 4 Hz, respectively.

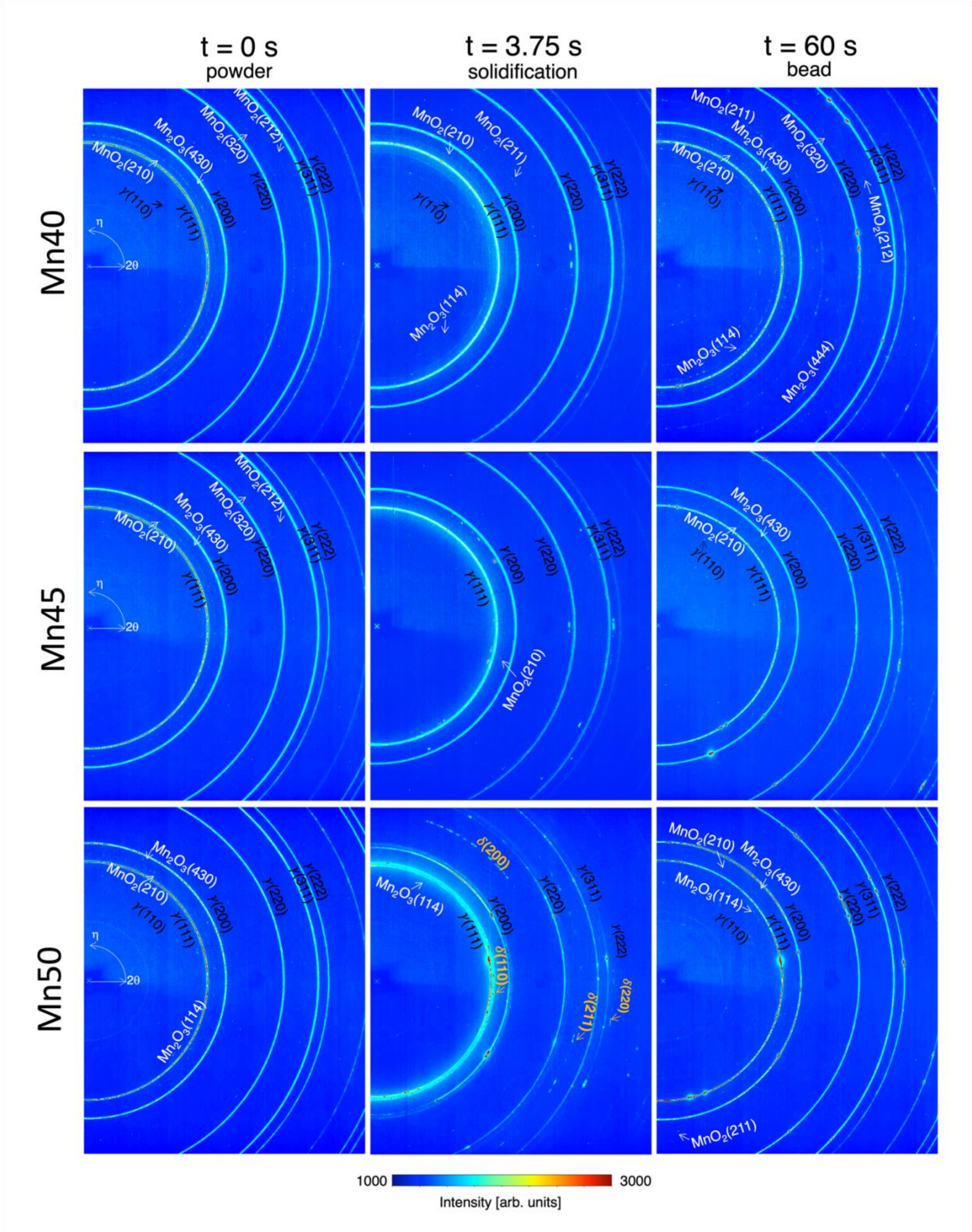

Supplementary Fig. 2. GE detector images of XRD patterns in powder, solidification, and bead for Mn40, Mn45, and Mn50. All rings corresponding to fcc (γ) are annotated in black, those

corresponding to bcc (δ) in yellow, and manganese oxides (MnO_2 and Mn_2O_3) in white. In Mn40 and Mn45, diffracted rings with higher intensities all correspond to the fcc phase throughout the process. In Mn50, all continuous rings correspond to the γ phase from powder diffraction (t = 0 s) as well. However, at the beginning of solidification (t = 3.75 s), additional diffraction spots emerge that correspond to the δ phase. These spots quickly disappear, and after cooling (t = 60 s), only the γ-fcc and manganese oxide rings remain.

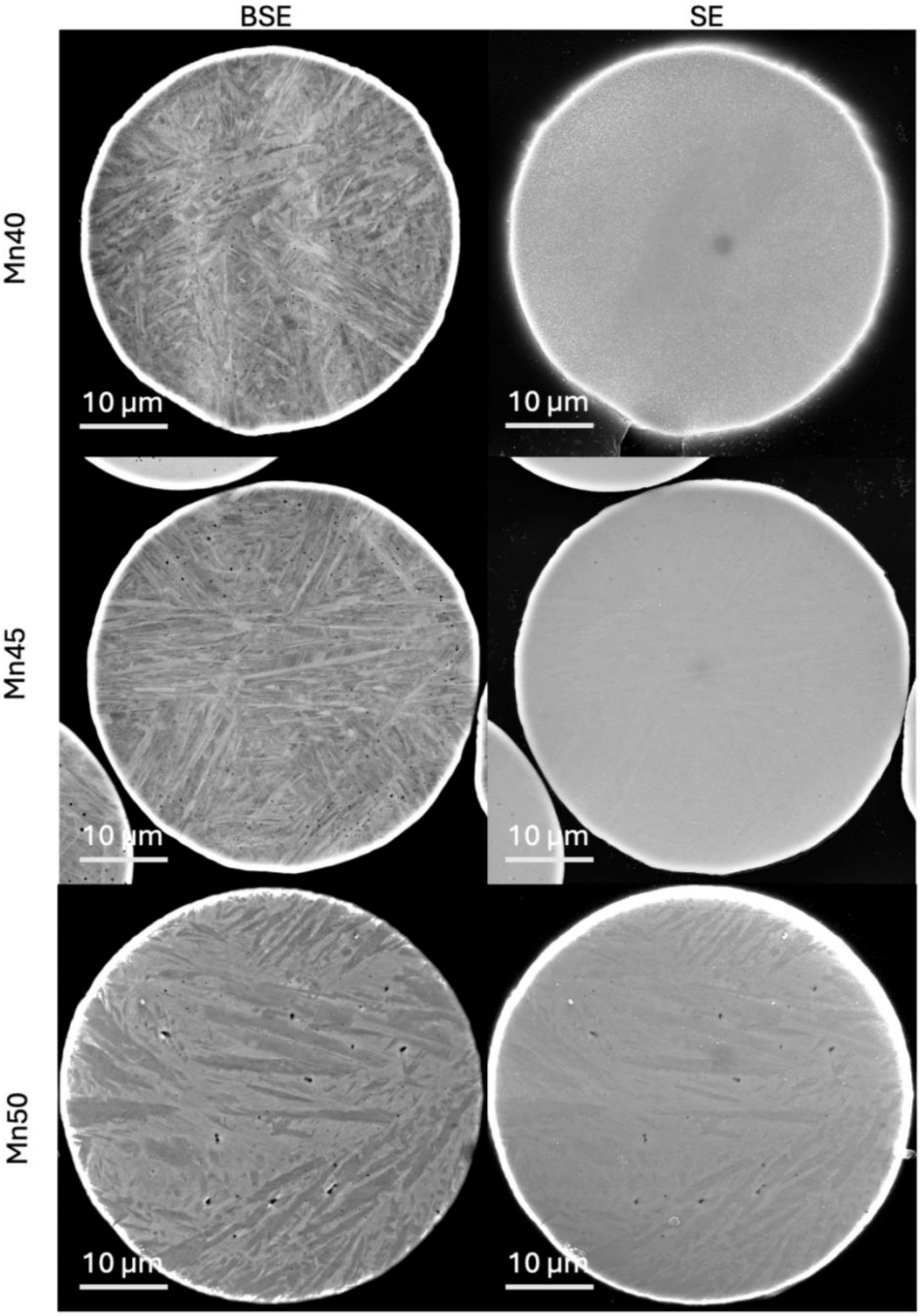

Supplementary Fig. 3. Backscatter electron (BSE) and secondary electron (SE) images of the cross-sections of representative powder particles of Mn40, Mn45, and Mn50.

Supplementary Table 1: Chemical composition of powder and as-printed FeMnCoCr measured via SEM-EDS. Mean and one standard deviation of five point-analyses are reported.

Composition (at.%)						
	Mn40		Mn45		Mn50	
	Powder	Print	Powder	Print	Powder	Print
Fe	40.6 ± 0.3	39.5 ± 1.5	36.7 ± 1.1	36.1 ± 0.9	31.7 ± 0.4	31.1 ± 1.5
Mn	40.0 ± 0.2	41.2 ± 1.3	43.6 ± 1.7	45.3 ± 0.6	48.8 ± 0.2	49.9 ± 1.1
Co	10.0 ± 0.2	9.9 ± 0.4	10.1 ± 0.2	9.2 ± 0.2	10.0 ± 0.1	9.8 ± 0.5
Cr	9.4 ± 0.3	9.0 ± 0.4	9.6 ± 0.5	9.4 ± 0.5	9.6 ± 0.2	9.2 ± 1.0

Supplementary Table 2: Composition of minor elements in FeMnCoCr powders.

Composition (wt.%)			
	Mn40	Mn45	Mn50
O	0.0574	0.0533	0.0364
N	0.0013	0.0011	0.0011
